# 1α,25(OH)_2_D_3_ Radiosensitizes Cancer Cells by Activating the NADPH/ROS Pathway

**DOI:** 10.3389/fphar.2020.00945

**Published:** 2020-08-07

**Authors:** Min-Tao Ji, Jing Nie, Xue-Fei Nie, Wen-Tao Hu, Hai-Long Pei, Jian-Mei Wan, Ai-Qing Wang, Guang-Ming Zhou, Zeng-Li Zhang, Lei Chang, Bing-Yan Li

**Affiliations:** ^1^ Department of Nutrition and Food Hygiene, Soochow University of Public Health, Suzhou, China; ^2^ State Key Laboratory of Radiation Medicine and Protection, School of Radiation Medicine and Protection, Collaborative Innovation Centre of Radiological Medicine of Jiangsu Higher Education Institutions, Soochow University, Suzhou, China

**Keywords:** 1α,25(OH)_2_D_3_, reactive oxygen species, radiosensitivity, NADPH oxidase, vitamin D receptor

## Abstract

The radioresistance of tumors affect the outcome of radiotherapy. Accumulating data suggest that 1α,25(OH)_2_D_3_ is a potential anti-oncogenic molecule in various cancers. In the present study, we investigated the radiosensitive effects and underlying mechanisms of 1α,25(OH)_2_D_3_ *in vitro* and *in vivo*. We found that 1α,25(OH)_2_D_3_ enhanced the radiosensitivity of lung cancer and ovarian cancer cells by promoting the NADPH oxidase-ROS-apoptosis axis. Compared to the group that only received radiation, the survival fraction and self-renewal capacity of cancer cells treated with a combination of 1α,25(OH)_2_D_3_ and radiation were decreased. Both apoptosis and ROS were significantly increased in the combination group compared with the radiation only group. Moreover, N-acetyl-L-cysteine, a scavenger of intracellular ROS, reversed the apoptosis and ROS induced by 1α,25(OH)_2_D_3_, indicating that 1α,25(OH)_2_D_3_ enhanced the radiosensitivity of cancer cells *in vitro* by promoting ROS-induced apoptosis. Moreover, our results demonstrated that 1α,25(OH)_2_D_3_ promoted the ROS level *via* activating NADPH oxidase complexes, NOX4, p22^phox^, and p47^phox^. In addition, knockdown of the vitamin D receptor (VDR) abolished the radiosensitization of 1α,25(OH)_2_D_3_, which confirmed that 1α,25(OH)_2_D_3_ radiosensitized tumor cells that depend on VDR. Similarly, our study also evidenced that vitamin D_3_ enhanced the radiosensitivity of cancer cells *in vivo* and extended the overall survival of mice with tumors. In summary, these results demonstrate that 1α,25(OH)_2_D_3_ enhances the radiosensitivity depending on VDR and activates the NADPH oxidase-ROS-apoptosis axis. Our findings suggest that 1α,25(OH)_2_D_3_ in combination with radiation enhances lung and ovarian cell radiosensitivity, potentially providing a novel combination therapeutic strategy.

## Introduction

Malignant cancer is a disease with one of the highest mortality rates in the world. At present, the general treatment for malignant cancer is based on radical surgery, radiotherapy, and chemotherapy (Miller et al., 2016). Although significant progress has been made in the past two decades, there are still some types of cancers that are insensitive to radiotherapy, which limits the treatment options for malignant cancers (Barker et al., 2015). The current challenge is to find a novel strategy to increase the radiosensitivity of malignant cancers.

Lung cancer is one of the most malignant cancers and has high morbidity and mortality worldwide, of which approximately 85% is non-small cell lung cancer. Despite different treatment strategies, the five-year survival rate of patients has improved slightly and remains at 4-17%(Hanahan and Weinberg, 2011; Fehrenbacher et al., 2016; Addeo, 2017; Hirsch et al., 2017). Similarly, ovarian cancer is also a malignancy tumor with high mortality (Miller et al., 2016), due to the existence of ovarian cancer stem cells (Brabletz et al., 2005; Aguilar-Gallardo et al., 2012; Flesken-Nikitin et al., 2013).

Radiotherapy plays a significant role in the treatment of tumors. Typically, there are two ways to kill cancer cells using radiotherapy. The first way is to break the DNA of cancer cells using high-intensity radiation. The second way is to generate a number of reactive oxygen species (ROS) to induce apoptosis by ionizing the water in cancer cells (Barker et al., 2015). Accumulating evidence has indicated that ROS may be a novel target for increasing radiosensitivity (Zou et al., 2017). In breast cancer, cordycepin is found to increase the radiosensitivity of cancer cells by increasing ROS (Dong et al., 2019). The mitochondrial and NADPH oxidase-derived ROS, combined with the radiotherapy, may increase the radiosensitivity of cancer cells (Chen et al., 2019; Mortezaee et al., 2019). Increasing ROS may therefore be an effective strategy for promoting radiosensitivity.

Several clinical studies and experimental studies support that 1α dihydroxyvitamin D [1α,25(OH)_2_D_3_] possesses anti-tumor actions in various cancers, such as breast-, colon-, and ovarian cancer (Larriba and Munoz, 2005; Pervin et al., 2013; Feldman et al., 2014; Griffin and Dowling, 2018). Additionally, low 25(OH)D serum was associated with higher ovarian cancer susceptibility and a poor prognosis for patients with lung cancer (Ong et al., 2016; Akiba et al., 2018). Furthermore, 1α,25(OH)_2_D_3_ and its analogue EB1089, have been shown to improve the radiosensitivity of cancer cells (Sundaram and Gewirtz, 1999; Demasters et al., 2006; Bristol et al., 2012), but the underlying mechanism is unclear. In this study, we shed light on the mechanism of how 1α,25(OH)_2_D_3_ sensitizes cancer cells to radiation, and test this both *in vitro* and *in vivo*. We found that 1α,25(OH)_2_D_3_ enhances the radiosensitivity of lung cancer and ovarian cancer cells that depend on VDR by promoting the NADPH oxidase-ROS-apoptosis axis.

## Materials and Methods

### Cell Culture and Reagents

Human ovarian epithelial adenocarcinoma cell line SKOV3 and lung cancer cell line A549 were obtained from the Type Culture Collection of the Chinese Academy of Sciences (Shanghai, China), and maintained in RPMI medium 1640 (Sigma-Aldrich Chemie GmbHFBS, Steinheim, Germany) with 10% fetal bovine serum (FBS, Sigma-Aldrich), 100 U/mL penicillin, and 100 μg/mL streptomycin (Beyotime Biotechnology, Shanghai, China) in a humidified atmosphere of 5% CO_2_ at 37°C. The pre-treatment time for 100 nM 1α,25(OH)_2_D_3_ for cells in the 1640 medium was 48 h, and the 1α,25(OH)_2_D_3_ and medium were changed every 24 h (in the dark). 1α,25(OH)_2_D_3_ was purchased from Sigma (Sigma-Aldrich).

### Radiation Delivery *In Vitro*

Briefly, cancer cell lines were irradiated by different doses of X-ray *in vitro*. IR was delivered at a dose rate of 1.0 Gy per minute using RS-2000 Pro (Rad source, USA).

### Colony Formation Assay

A549 and SKOV3 cells were trypsinized and dissociated into single-cell suspensions for plating in 12-well plates or 60 mm culture dishes. The cells were treated with 1α,25(OH)_2_D_3_ for 48 h before X-ray (0, 1, 2, 4 Gy) radiation. After culturing for 14 days at 37°C, the colonies were fixed with 75% alcohol and stained with 0.3% methyl violet for 20 min at room temperature. Then the colonies were dissolved by glacial acetic acid and detected the absorbance value. The inhibition viability of 1 Gy, 2 Gy and 4 Gy were obtained by comparing with the absorbance of 0 Gy group.

### Assessment of Apoptosis Using Flow Cytometry

A549 and SKOV3 cells were treated with 100 nM 1α,25(OH)_2_D_3_ for 48 h, following 4 Gy X-ray radiation. After 24 h, the cells were harvested, washed with PBS, and incubated for 15 min at room temperature in a binding buffer containing Annexin V-FITC or Annexin V-PE and PI or 7-AAD (Jiangsu KeyGEN Bio TECH Corp., Ltd, China) before flow cytometry (FC500, Beckman Coulter, Inc., Brea, CA, USA). All the quadrants, except the left lower quadrant, were determined and analyzed by histograms.

### Assessment of Intracellular ROS Using Flow Cytometry

2,7-dichlorodihydrofluorescein diacetate (DCFH-DA) (Beyotime Biotechnology) was used to determine intracellular ROS levels. A549 and SKOV3 cells were treated with 100 nM 1α,25(OH)_2_D_3_ for 48 h, following 4 Gy X-ray radiation. After 24 h, cells were pre-incubated with 10 μM DCFH-DA for 30 min at 37°C. Mean fluorescence intensity was determined after washing the cells three times with PBS before flow cytometry.

### Assessment of Mitochondrial Membrane Potential (MMP) Using Flow Cytometry

Rhodamine 123 (Beyotime Biotechnology) was used to determine the MMP levels. In normal cells, Rho123 can enter the mitochondrial matrix through the mitochondrial transmembrane potential and the fluorescence intensity decreases or disappears. When it comes to apoptosis and mitochondrial membrane integrity, Rho123 is released outside the mitochondria and expresses a strong green fluorescence (Hwang et al., 2007; de Menezes et al., 2019). Cells were treated under the same conditions as described above and were then pre-incubated with 10 μM Rho123 for 30 min at 37°C. Mean fluorescence intensity was determined for cells after three washes with PBS before flow cytometry. The software used for evaluating the flow cytometry was CXP Analysis, which is the part of the FC500 instrument.

### Sphere-Formation Assay

A549 and SKOV3 cells tested in the control, 1α,25(OH)_2_D_3_, radiation, and combination groups were trypsinized and dissociated into single-cell suspensions. Subsequently, 1,000 cells/well were plated in 96-well plates and cultured in serum-free medium for 10 days. The spheres with a minimum size of 50 μm were counted under the brightfield microscope (CKX41F; Olympus Corporation, Tokyo, Japan) equipped with a digital camera. The sphere-formation rate was expressed as the following formula: (Number of spheres formed/number of plated cells) x 100.

### Limited Dilution Assay

A549 and SKOV3 cells were dissociated into single-cell suspensions as described above. Subsequently, 250, 125, 50, 25, 12, or 6 cells/hole were plated into 96-well plates and cultured in serum-free medium for 10 days. The spheres with a minimum size of 50 μm were counted under the brightfield microscope equipped with a digital camera. The hole of the neurosphere rate was expressed as the following formula: (Number of holes with neurosphere formation/number of holes plating the cells) x 100%.

### Protein Sample Preparation and Western Blotting

Cancer cells were lysed in protein lysis buffer containing protease and phosphatase inhibitors (Roche, Mannheim, Germany) for 30 min at 4°C and were then centrifuged at 13,000×g at 4°C for 15 min for the collection of total cell lysates. The protein concentrations were analyzed using Western blot with methods we previously described (Ji et al., 2019). VDR (1:1000, CST:12550S), p47^phox^ (1:1000, CST:4312S), and p22^phox^ (1:1000, Abcam: ab75941) were diluted in 5% BSA and incubated overnight at 4°C. GAPDH was determined as a loading control.

### Knockdown of VDR With shRNA

Control shRNA against VDR were procured from Shanghai Genechem (Shanghai, China). SKOV-3 and A549 cells were transfected with the shRNA using Polybrene transfection reagent from Shanghai Genechem according to the manufacturer’s instructions. The cells were incubated for 24 h, followed by 2 μg/mL of puromycin selecting for 24 h. The selected stable cell line expressing shVDR was maintained in RPMI medium 1640 with 2 μg/mL puromycin. shVDR-1: CCGGGTCATCATGTTGCGCTCCAATCTCGAGATTGGAGCGCAACATGATGACTTTTTG. shVDR2: CCGGCCTCCAGTTCGTGTGAATGATCTCGAGATCATTCACACGAACTGGAGGTTTTTG.

### RNA Isolation and Real-Time Quantitative RT-PCR Analysis

Total RNA was isolated using RNAiso Plus (Takara Biomedical Technology (Beijing) Co., Ltd, China) and 1 µg of RNA was reverse transcribed with the PrimeScript^™^ RT Master Mix (Perfect Real Time) (Takara Biomedical Technology) according to the manufacturer’s protocol. qRT-PCR was performed using the Applied Biosystems 7500 Real-Time PCR System (Thermo Fisher Scientific, Inc. Waltham, MA, USA). Expression of the genes of interest was analyzed using the GAPDH gene as the internal control and the ΔΔCt method. Real time primer information is provided in **Table 1**.

**Table 1:** Primer sequences for reverse transcription-quantitative polymerase chain reaction.

Gene	Forward Sequence (5’-3’)	Reverse Sequence (5’-3’)
p47^phox^	CTGACGAGACGGAAGACCC	GGACGGAAAGTAGCCTGTGA
NOX4	GCAAGATACCGAGATGAG	ACAGTACAGGCACAAAGG
GAPDH	TTGATGGCAACAATCTCCAC	CGTCCCGTAGACAAAATGGT
VDR	GGAGAAAACACTTGTAAGTTGCT	TGGTCAGGTTGGTCTCGAACT
p22^phox^	CATTGTGGCGGGCGTGTT	TCCTCGCTGGGCTTCTTGC

### Tumorigenesis

Female BALB/c nude mice (4–6 weeks, 18–20 g) were purchased from Soochow University Laboratory Animal Center (Suzhou, China). Mice were provided with water and food *ad libitum* and were housed with five animals per cage. Controlled environmental conditions were temperature at 21 ± 2°C, relative humidity at 55 ± 5% and a 12:12 h light–dark cycle. All surgical procedures and care provided to the animals were approved by the Institutional Animal Care and Use Committee (approval number ECSU–201800049). A total of 2x10^7^ OVCAR8-luciferase cells in 200 µl cell suspension mixed with Matrigel (1:1; BD Biosciences; cat. no. 356234) were injected subcutaneously into these mice. The subcutaneous model was used to evaluate the effect of vitamin D_3_ on the radiosensitivity of cancer cells. When the tumor volume was around 40 mm^3^ at 3 weeks post injection, mice were entered into the study. All mice were randomized into four groups as follows: (1) control (N=5); (2) vitamin D_3_ (N=5); (3) local ionizing radiation (IR) (N=10); and (4) vitamin D_3_ + local IR (N=10). The mice in the vitamin D_3_–treatment group were injected with a single dose of vitamin D_3_ (1,000 IU/week) intramuscularly. The luciferase signals were detected every week. Three weeks after cell injection, the mice were irradiated with 15Gy X-ray by X-RAD SmART (Precision X-Ray Inc., USA). The animals were sacrificed 6–8 weeks after cell injection, when the tumor nodules were identified on their body surface. The sacrificing of mice were performed using 3% pentobarbital sodium (150 mg/kg), followed by cervical dislocation to ensure death.

### Immunohistochemical Staining

Immunohistochemical analyses were performed using method previously described (Wang et al., 2017). Tumor tissue samples were harvested, fixed in 4% paraformaldehyde/PBS, dehydrated, embedded in paraffin blocks, and cut into 5 μm thick sections. Deparaffinized tissue sections were rehydrated and stained using specific antibodies for Ki-67 or TUNEL, before incubating and staining with biotinylated secondary antibodies. Signal intensity was determined with an avidin-biotin horseradish peroxidase complex and 3,3’-diaminobenzidine (BD Biosciences) as the chromogen. Representative photographs were taken using an Olympus IX73 microscope (Olympus America, Melville, NY, USA) at 400× magnification, after analyzing all slides

### Statistical Analysis

Experiments were performed independently at least three times, and the resulting data represented mean ± standard error of the mean. Statistical comparisons were made for two groups using Student’s *t*-test. We also used one-way analysis of variance with repeated measures, followed by *post hoc* comparisons using Tukey’s multiple paired comparison test, to define differences between groups for statistical significance. *P* < 0.05 was defined as statistically significant. GraphPad Prism 7 software (San Diego, CA, USA) was used for all analyses in this study.

## Results

### 1α,25(OH)_2_D_3_ Enhanced the Radiosensitivity of Human Ovarian Cancer and Lung Cancer Cells

**Figure 1:**
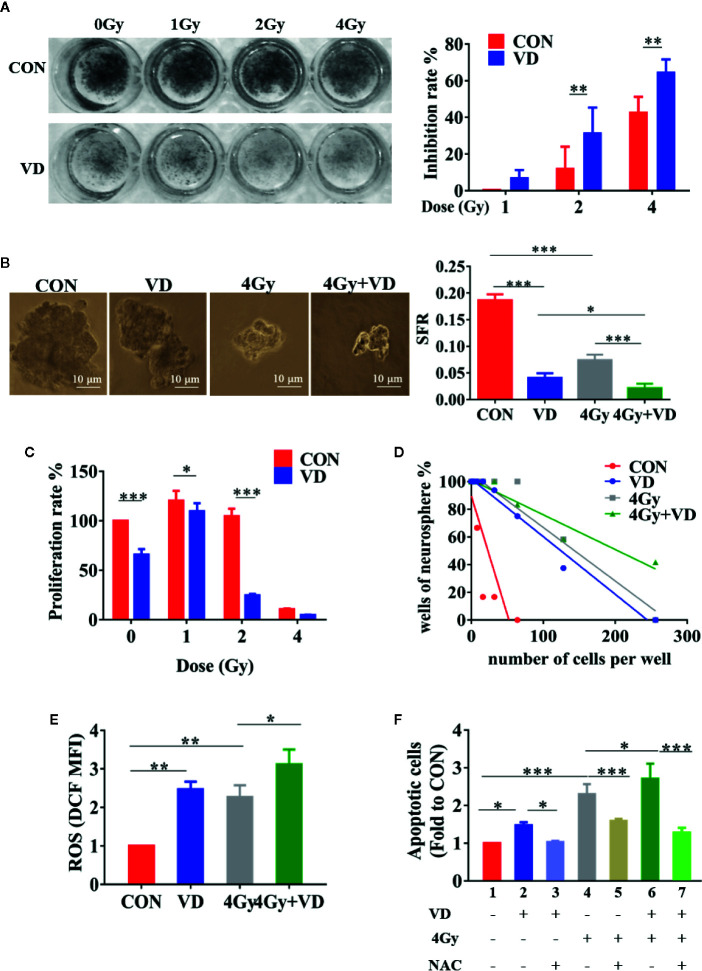
1α,25(OH)_2_D_3_ enhanced the radiosensitivity of human ovarian cancer and lung cancer cells by ROS-induced apoptosis. **(A, B)** Results of colony formation assay and sphere formation assay in A549 cancer cells. **(C)** Results of colony formation assay in SKOV3 cancer cells. **(D)** Results of limited dilution assay in A549 cancer cells. **(E)** Effects of 1α,25(OH)_2_D_3_ and radiation on ROS of A549 cancer cells, related to . **(F)** Combined effects of 1α,25(OH)_2_D_3_, radiation and NAC on apoptosis of A549 cancer cells, related to . Line 1 represents the control group, line 2 represents the 1α,25(OH)_2_D_3_ alone group, Line 3 represents the 1α,25(OH)_2_D_3_ and NAC group, Line 4 represents the radiation alone group, Line 5 represents the radiation and NAC group, Line 6 represents the 1α,25(OH)_2_D_3_ and radiation group, Line 7 represents the 1α,25(OH)_2_D_3_, radiation, and NAC group. Data represents the Mean ± SD, *p < 0.05, **p < 0.01, ***p < 0.001, n=3.

In the present study, the colony formation and sphere formation assays were used to evaluate the effects of 1α,25(OH)_2_D_3_ on the radiosensitivity of the cancer cells. With radiation alone, the colony formation in A549 cells was decreased in a dose-dependent manner. At 4 Gy radiation, 43.19% of cell proliferation was inhibited compared with non-radiated cells. Treatment with 100 nM 1α,25(OH)_2_D_3_ combined with radiation led to significantly less colony formation in comparison with radiated cells (**Figure 1A**). Although 4 Gy radiation or 1α,25(OH)_2_D_3_ treatment alone could decrease sphere formation to 62% and 78%, treatment with 1α,25(OH)_2_D_3_ combined with radiation resulted in an 89% reduction compared to non-radiated cells. This result shows that 1α,25(OH)_2_D_3_ and radiation has a significantly synergistic effect to kill cancer cells (**Figure 1B**). The colony formation assay demonstrated that 1α,25(OH)_2_D_3_ also significantly decreased the colony formation when SKOV3 cells were irradiated at 0, 1, 2 and 4 Gy X-ray (**Figure 1C**).

The limited dilution assay was generally used to determine the tumorigenicity *in vitro*. By using this assay to test A549 and SKOV3, we found that radiation or 1α,25(OH)_2_D_3_ treatment alone decreased the tumorigenicity. Moreover, the treatment with 1α,25(OH)_2_D_3_ for 48 h, combined with radiation, remarkably reduced tumorigenicity compared to irradiated-only or 1α,25(OH)_2_D_3_ treatment alone (**Figure 1D** and **S1A**). Altogether, the results suggest that 1α,25(OH)_2_D_3_ enhances the radiosensitivity of human ovarian and lung cancer cells.

### 1α,25(OH)_2_D_3_ Promoted ROS-Induced Apoptosis in Irradiated Cancer Cells

Cancer radiotherapy largely depends on ROS generation to destroy malignant cells by inducing apoptosis. In the present study, the exposure cells to either 1α,25(OH)_2_D_3_ or 4 Gy X-ray resulted in elevated levels of ROS, and both also induced apoptosis, compared to non-treated A549 cells. Interestingly, the treatment with 1α,25(OH)_2_D_3_ before radiation further produced ROS and induced apoptosis, which significantly increased apoptosis and the ROS on the basis of radiation (**Figures 1E, F** and **S1B**). Moreover, we also detected the autophagy and senescence (**Figures S3** and **S4**). Of note, 1α,25(OH)_2_D_3_ or X-ray per se can promote autophagy, but there is no synergistic effect between 1α,25(OH)_2_D_3_ and X-ray.

N-acetylcysteine (NAC) is not only a scavenger of intracellular ROS (Halasi et al., 2013), but also an antioxidant known to lack direct radioprotective properties (Grdina et al., 2002). To elucidate the role of ROS in inducing apoptosis, NAC was used to decrease ROS produced by 1α,25(OH)_2_D_3_. Indeed, the administration with NAC for 27 h rescued 1α,25(OH)_2_D_3_-induced ROS to control levels (**Figure S1C**). Treating cells with 1α,25(OH)_2_D_3_ and NAC could significantly decrease the apoptosis compared to 1α,25(OH)_2_D_3_ treatment only, indicating that 1α,25(OH)_2_D_3_ may promote apoptosis by producing ROS (**Figure 1F**, line 3 vs line 2 and **Figure 1E**). Compared to irradiated-cells alone, the combined NAC and irradiation treatment decreased apoptosis (**Figure 1F**, line 5 vs line 4). But this apoptosis was higher than non-irradiated cells, suggesting that both elevating ROS and DNA damage were accountable for the apoptosis induced by radiation (**Figure 1F**, line 5 vs line 1). Moreover, apoptosis in the combined radiation and 1α,25(OH)_2_D_3_ group was dramatically decreased when the ROS was removed by NAC (**Figure 1F**, line 7 vs line 6). These indicated that 1α,25(OH)_2_D_3_ enhanced the radiosensitivity of cancer cells by ROS-induced apoptosis.

### The Enhancing Radiosensitivity of 1α,25(OH)_2_D_3_ Depends on VDR

**Figure 2:**
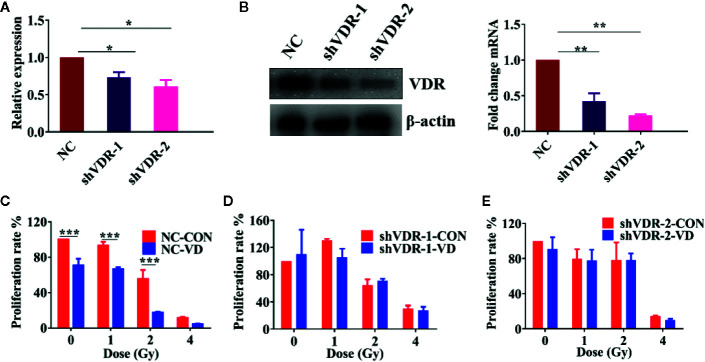
The enhancing radiosensitivity of 1α,25(OH)_2_D_3_ depends on VDR. **(A, B)** The mRNA and protein levels of VDR were examined when treated with shNC and shVDR in SKOV3 cancer cells. **(C–E)** Colony formation assay was examined in SKOV3 cancer cells when treated with shNC and shVDR. The data represents the Mean ± SD, *p < 0.05, **p < 0.01, ***p < 0.001, n = 3.

1α,25(OH)_2_D_3_ exhibits its biological functions mainly through binding to its receptor (Vitamin D Receptor, VDR). To gain further insights into the radiosensitivity of 1α,25(OH)_2_D_3_, VDR was knocked down in A549 and SKOV3 cells by short hairpin RNAs (shVDR). Both mRNA and protein levels of VDR in SKOV3 and A549 cells were successfully suppressed, compared with the corresponding negative control (NC) (**Figures 2A, B** and **S2A, B**). Compared to irradiated-NC cells, 1α,25(OH)_2_D_3_ inhibited the colony formation rate of NC cells, indicating that 1α,25(OH)_2_D_3_ still enhanced the radiosensitivity of cancer cells (**Figures 2C** and **S2C**). However, 1α,25(OH)_2_D_3_ did not decrease the colony formation rate in VDR knock down (shVDR) cells followed by X-ray irradiation, suggesting that VDR knockdown could not sensitize 1α,25(OH)_2_D_3_-treated cells to radiation (**Figures 2D, E** and **S2D, E**). These results indicated that the enhancement of radiosensitivity in cancer cells, by 1α,25(OH)_2_D_3_, depends on VDR.

**Figure 3:**
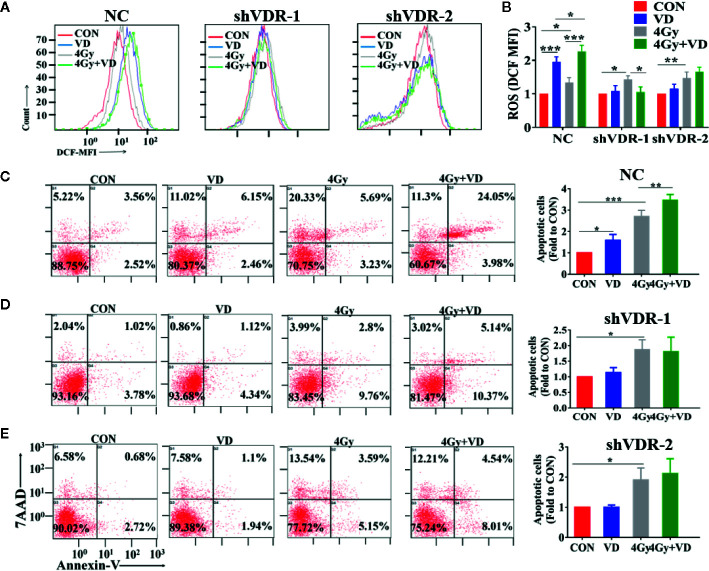
Effects of 1α,25(OH)_2_D_3_ on ROS and apoptosis depends on VDR. **(A, B)** ROS was examined in A549 cancer cells when treated with shNC and shVDR. **(C–E)** Apoptosis percentages was examined in A549 cancer cells when treated with shNC and shVDR. The data represents the Mean ± SD, *p < 0.05, **p < 0.01, ***p < 0.001, n = 3.

To test whether the ROS-induced apoptosis by 1α,25(OH)_2_D_3_ is associated with VDR, we also detected the ROS and apoptosis percentages in VDR knockdown cells. As shown in **Figures 3A–C**, compared with single irradiated NC cells, 1α,25(OH)_2_D_3_ still promoted the ROS levels, and increased the apoptosis percentages in combination with radiation. However, when it comes to VDR knockdown cells, the effects of 1α,25(OH)_2_D_3_ on increasing ROS and apoptosis percentages were disappeared (**Figures 3A, B, D, E**). These results demonstrated that 1α,25(OH)_2_D_3_ promoted cancer cells ROS and apoptosis by binding to VDR. Together with above results, this suggests that 1α,25(OH)_2_D_3_ enhances the radiosensitivity by the VDR-ROS-apoptosis axis.

### 1α,25(OH)_2_D_3_ Enhanced the Radiosensitivity of Cancer Cells by Activating the NADPH Oxidase-ROS-Apoptosis Axis

**Figure 4:**
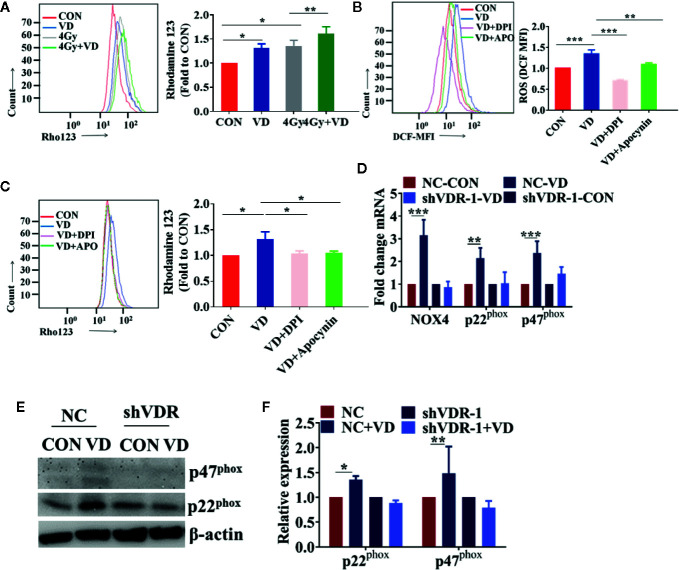
1α,25(OH)_2_D_3_ enhanced the radiosensitivity of cancer cells by activating the NADPH oxidase-ROS-apoptosis axis. **(A)** MMP level was examined by Rho 123 in A549 cancer cells. **(B)** ROS was examined in A549 cancer cells when treated with DPI and Apocynin. **(C)** MMP level was examined by Rho 123 in A549 cancer cells treated with DPI and Apocynin. **(D)** The mRNA levels of NOX4, p22^phox^ and p47^phox^ were examined in shNC and shVDR A549 cancer cells treated with 1α,25(OH)_2_D_3_. **(E, F)** The protein levels of p22^phox^ and p47^phox^ were examined in shNC and shVDR A549 cancer cells treated with 1α,25(OH)_2_D_3_. The data represents the Mean ± SD, *p < 0.05, **p < 0.01, ***p < 0.001, n = 3.

Given that ROS mainly originated from mitochondria or NADPH oxidase, the origin of ROS induced by 1α,25(OH)_2_D_3_ was investigated in the present study. First, the mitochondrial membrane potential level was detected, and it was found that 1α,25(OH)_2_D_3_ enhanced the mitochondrial injury on the basis of radiation (**Figure 4A**). This indicated that ROS generated by 1α,25(OH)_2_D_3_ can accumulate in the mitochondria. Meanwhile, DPI and apocynin, the NADPH oxidase specific inhibitors, were used to detect the changes of ROS generated by 1α,25(OH)_2_D_3_. Interestingly, DPI and apocynin can both rescue ROS levels induced by 1α,25(OH)_2_D_3_ (**Figure 4B**). These results implied two hypotheses, that 1α,25(OH)_2_D_3_ may induce ROS both in mitochondrial and NADPH oxidase; and that 1α,25(OH)_2_D_3_ may induce ROS in NADPH oxidase, then ROS is delivered to mitochondria and injures the mitochondria. To test these hypotheses, mitochondrial membrane potential on the basis of removing ROS by DPI and apocynin was detected. The results showed that the injury induced by 1α,25(OH)_2_D_3_ disappeared (**Figure 4C**), in agreement with the second hypothesis. To further investigate whether 1α,25(OH)_2_D_3_ could activate the NADPH oxidase, the mRNA and protein levels of the NADPH oxidase complexes, NOX4, p22^phox^ and P47^phox^, were detected. The results indicated that 1α,25(OH)_2_D_3_ can activate the NADPH oxidase complexes by binding to VDR in NC cells but not the VDR knockdown cells (**Figures 4D–F**). Together these results demonstrate that 1α,25(OH)_2_D_3_ enhances the radiosensitivity by binding to VDR and activating the NADPH oxidase-ROS-apoptosis axis.

### Vitamin D_3_ Enhanced the Radiosensitivity of Ovarian Cancer *In Vivo*

**Figure 5:**
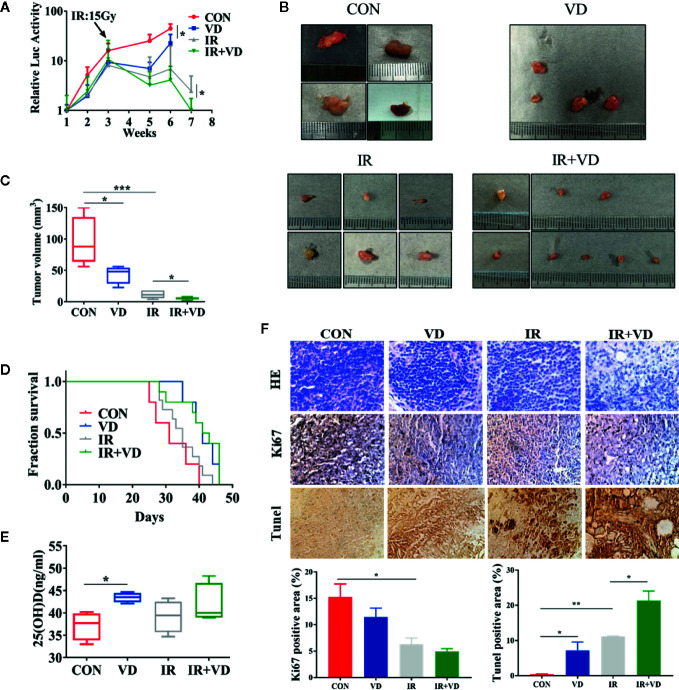
Vitamin D_3_ enhanced the radiosensitivity of ovarian cancer *in vivo*. **(A)** The relative luciferase signals were detected in nude mice of control, vitamin D_3_, radiation, and combined groups. **(B, C)** Image and the volume of tumors. **(D)** The overall survival of nude mice. **(E)** Serum 25(OH)D levels of nude mice. **(F)** Representative images of tumors stained for HE, Ki67 and Tunel. The data represents the Mean ± SD, *p < 0.05, **p < 0.01, ***p < 0.001, n = 3.

To determine whether vitamin D increases the radiosensitivity of cancer cells ***in vivo***, we created a subcutaneous xenograft tumor mode using female nude mice. Vitamin D_3_ alone and local IR could inhibit growth of xenograft tumors that arose from OVCAR8 cells. But the combination of IR and Vitamin D_3_ shows joint effects in killing the cancer cells both on relative luciferase, which is a tracing marker to value the tumor growth (**Figure 5A**), and final tumor size and volume (**Figures 5B, C**). Meanwhile, vitamin D_3_ treatment improved overall survival from 31 to 41 days (vitamin D_3_ group vs control group), combined with IR it further improved overall survival from 35 to 42 days (vitamin D_3_ and IR group vs. IR group) (**Figure 5D**). The 25(OH)D levels in serum increased in vitamin D_3_ treatment, but the combination of Vitamin D_3_ and IR seems not to have further increased 25(OH)D levels (**Figure 5E**). We also examined the expression of the cell proliferation marker Ki-67 and the apoptosis signature, TUNEL positive cells, in these tumors. Immunohistochemical staining showed that compared with the radiation group, apoptosis percentages in the vitamin D_3_ and radiation combined group significantly increased (**Figure 5F**), which further indicated that vitamin D_3_ can increase the radiosensitivity of tumors *in vivo*. Altogether, considering these results, we demonstrated that vitamin D_3_ enhanced the radiosensitivity of cancer cells *in vivo* and extended the overall survival of mice with tumors.

## Discussion

Radiotherapy is one of the most effective and frequently used tumor treatments, however, both the radioresistance of tumors and the damage caused to healthy tissues nearby tumors under high-dose ionizing radiation, limit the use of radiotherapy. Natural and synthetic vitamin D compounds have been proven as anticancer agents (Larriba and Munoz, 2005; Pervin et al., 2013; Feldman et al., 2014; Griffin and Dowling, 2018 **)**. It has been reported that vitamin D and vitamin D analogs radiosensitized breast cancer and lung cancer cells through altering the nature of the autophagy, converting it from a protective form to a cytotoxic form *in vitro (* Sundaram and Gewirtz, 1999; Demasters et al., 2006; Bristol et al., 2012 **)**. In the present study, we confirmed that 1α,25(OH)_2_D_3_ enhances the radiosensitivity of human ovarian and lung cancer cells by activating NADPH oxidase-ROS-apoptosis axis *in vitro*. Moreover, knockdown of VDR abolishes the radiosensitization of 1α,25(OH)_2_D3, which implies that 1α,25(OH)_2_D_3_ exhibits a radiosensitizer that depends on VDR. Our study also evidenced that vitamin D_3_ enhances the radiosensitivity of cancer cells *in vivo* and extends the overall survival of mice with tumors.

1α,25(OH)_2_D_3_ not only affects the metabolism of calcium and phosphorus, but also inhibits the proliferation, invasion, and migration of cancers, including colon-(Pendas-Franco et al., 2008; Leyssens et al., 2013; Klampfer and Vitamin, 2014; Chen et al., 2015), prostate-(Leyssens et al., 2013), breast-(Wahler et al., 2015), ovarian-(Lungchukiet et al., 2015), and lung cancer (Tao et al., 2017). Epidemiological studies have also observed that high levels of 25(OH)D in serum can reduce the risk of many types of cancers, and improve the survival of ovarian cancer patients (Webb et al., 2015; Granato et al., 2016; Ong et al., 2016; Akiba et al., 2018; Estebanez et al., 2018; Goulao et al., 2018). Moreover, 1α,25(OH)_2_D_3_ and its analogue EB1089, improved the radiosensitivity of cancer by inducing autophagy and inhibiting DNA repair in breast cancer (Sundaram and Gewirtz, 1999; Demasters et al., 2006; Bristol et al., 2012). Meanwhile, 1α,25(OH)_2_D_3_ enhanced radiosensitivity of prostatic cancer by inhibiting MnSOD and mitochondrial antioxidant reactions (Xu et al., 2007). Here, our study evidenced that 1α,25(OH)_2_D_3_ enhances the radiosensitivity of human lung and ovarian cancer cells *via* activating NADPH oxidase-ROS-apoptosis axis.

Interestingly, there were inconsistencies with the notion of the effect of 1α,25(OH)_2_D_3_ on oxidative stress. There are some studies that show that 1α,25(OH)_2_D_3_ acts as an antioxidant in normal epithelial cells (Dai et al., 2018; Tang et al., 2018). Some other studies also indicate that 1α,25(OH)_2_D_3_ promotes DNA repair when human foreskin (BJs) and lung (IMR90) fibroblasts were induced by Ras (Bao et al., 2008; Graziano et al., 2016; Mark et al., 2016). Meanwhile, other studies suggest that 1α,25(OH)_2_D_3_ enhances oxidative stress during the early progression of breast cancer by inhibiting the activity of pyruvate carboxylase (Wilmanski et al., 2017). Piotrowska A et al. report that 1α,25(OH)_2_D_3_ promotes the oxidative damage of H_2_O_2_ on human keratinocytes (Piotrowska et al., 2016). In the present study, consistent with enhancement of oxidative stress in breast cancer (Wilmanski et al., 2017), we found that 1α,25(OH)_2_D_3_ promoted ROS levels in lung and ovarian cancer cells.

Of note, 1α,25(OH)_2_D_3_ plays completely different roles in normal cells and cancer cells. On one hand, it maintains the genomic stability of normal cells. First, it promotes the expression of DNA repair protein RAD50 and ATM, and reduces the genetic toxicity caused by chemicals (Ting et al., 2012); second, 1α,25(OH)_2_D_3_ promotes SOD, GSH and reduces glutathione reductase to decrease oxidant damage (Choi and Jung, 2017); third, it prevents malignant transformation of prostate epithelial cells by inhibiting oxidant damage (Bao et al., 2008). Most importantly, 1α,25(OH)_2_D_3_ protects the lung from radiation-induced injury by inducing the proliferation of type II lung cells and the synthesis of surfactant and reducing the vascular permeability caused by radiation (Yazici et al., 2011). On the other hand, 1α,25(OH)_2_D_3_ inhibits growth of cancer cells, which can be divided into the following aspects: inhibiting the MAPK and ERK pathways; inducing apoptosis by IGF-PI3K-AKT pathway (Deeb et al., 2007). Here we demonstrated that 1α,25(OH)_2_D_3_ could enhance radiosensitivity of cancer cells by promoting ROS-induced apoptosis. Therefore, all the above evidence indicates that 1α,25(OH)_2_D_3_ is a potential radiosensitizer, which enhances the radiosensitivity of cancer cells and which protects normal cells.

X-rays/γ-rays kill tumor cells *via* inducing direct interactions with biomolecules and indirectly causing radiolysis of water molecules within tumor cells to generate ROS (Barker et al., 2015), which can indirectly induce cell apoptosis. One way of reducing the radioresistance has been proposed to increase ROS generation (Lowe et al., 1994; Lotem et al., 1996; Pelicano et al., 2004; Azad et al., 2009). In this study, we found that 1α,25(OH)_2_D_3_ further increased the ROS level and apoptosis in cancer cells irradiated by X-ray. To address whether the apoptosis was induced by the overproduction of ROS by 1α,25(OH)_2_D_3_ (Zhang et al., 2015; Guo et al., 2017; Zhou et al., 2019), we detected the apoptosis of the cells which were eliminating the ROS production by NAC (Halasi et al., 2013). We found that the apoptosis was dramatically decreased upon NAC treatment. This result indicates that NAC abolished the enhancement of radiosensitivity caused by 1α,25(OH)_2_D_3_. Furthermore, our results for the first time evidenced that knockdown VDR impaired the radiosensitivity of 1α,25(OH)_2_D_3_ through ROS-induced apoptosis.

ROS can be derived from different sources, including the mitochondria electron transport chain, xanthine oxidase, the cytochrome P450 system, uncoupled nitric oxide synthase, and myeloperoxidase. ROS also mainly comes from two main parts, mitochondrial and NADPH oxidase after radiation (Bedard and Krause, 2007; Bhattacharyya et al., 2014; Cho et al., 2017; Pei et al., 2017; Kawamura et al., 2018; Sakai et al., 2018; Shimura et al., 2018; Mortezaee et al., 2019). To explore the origin of ROS induced by 1α,25(OH)_2_D_3_, both NADPH oxidase inhibitors, DPI and Apocynin, and the mitochondrial membrane potential were used to determine the source of ROS (Bedard and Krause, 2007). Our results indicated that 1α,25(OH)_2_D_3_ promoted ROS levels *via* activating NADPH oxidase complexes, NOX4, p22^phox^, and P47^phox^, but not in VDR knockdown cells. The overproduction of ROS was then delivered to mitochondria and caused damage to mitochondria, because of that the mitochondrial damage generated by 1α,25(OH)_2_D_3_ disappeared after inhibiting ROS. Altogether, our results demonstrate that 1α,25(OH)_2_D_3_ promotes the production of ROS by activating NADPH oxidase. Therefore, 1α,25(OH)_2_D_3_ exhibits a potential radiosensitizer by the overproduction of ROS, which is an important target of radiosensitivity in multiple cancers (Storch et al., 2016; Pajic et al., 2018).

In conclusion, 1α,25(OH)_2_D_3_ radiosensitizes cancer cells that depend on VDR, by activating the NADPH oxidase complex, which further increases the ROS level and induces apoptosis. Our findings evidence that 1α,25(OH)_2_D_3_ may be a potential radiosensitizer for a tumor combination therapeutic strategy.

## Data Availability Statement

The datasets generated for this study are available on request to the corresponding authors.

## Ethics Statement

The animal study was reviewed and approved by Institutional Animal Care and Use Committee of Soochow University.

## Author Contributions 

Conceptualization: M-TJ, X-FN, and B-YL. Methodology: M-TJ, JN, W-TH, H-LP, A-QW, and J-MW. Software: M-TJ, X-FN, and JN. Validation: JN, W-TH, and H-LP. Formal analysis: M-TJ and JN. Investigation: W-TH and H-LP. Resources: LC and B-YL. Data curation: M-TJ, X-FN, JN, LC, and B-YL. Writing—original draft preparation: M-TJ and X-FN. Writing—review and editing: M-TJ, LC, and B-YL. Visualization: JN, LC, and B-YL. Supervision: LC and B-YL. Project administration: LC and B-YL. Funding acquisition: B-Y.L, Z-LZ, and G-MZ.

## Funding 

This work was supported by the National Natural Science Foundation of China (No. U1832140, 81673151, 81372979) and the National Key R&D Program of China (No. 2018YFC0115704, 2018YFC0115705).

## Conflict of Interest

The authors declare that the research was conducted in the absence of any commercial or financial relationships that could be construed as a potential conflict of interest.

## Supplementary Material

Figure S1 1α,25(OH)_2_D_3_ enhanced the radiosensitivity of human ovarian cancer and lung cancer cells by ROS-induced apoptosis. **(A)** Results of limited dilution assay in SKOV3 cancer cells. **(B)** Effects of 1α,25(OH)_2_D_3_ and radiation on ROS of A549 cancer cells. **(C)** Effects of NAC to decrease the ROS generated by 1α,25(OH)_2_D_3_ in A549 cancer cells. **(D)** Combined effects of 1α,25(OH)_2_D_3_ and radiation on apoptosis of A549 cancer cells. Data represents the Mean ± SD, *p < 0.05, **p < 0.01, ***p < 0.001, n = 3.

Figure S2 The enhancing radiosensitivity of 1α,25(OH)_2_D_3_ depends on VDR. **(A, B)** The mRNA and protein levels of VDR were examined when treated with shNC and shVDR in A549 cancer cells. **(C-E)** Colony formation assay was examined in A549 cancer cells when treated with shNC and shVDR. The data represents the Mean ± SD, * p < 0.05, ** p < 0.01, *** p < 0.001, n=3.

Figure S3 The enhancing radiosensitivity of 1α,25(OH)_2_D_3_ does not depend on autophagy. **(A, B)** The protein levels of P62 were examined when treated with 1α,25(OH)_2_D_3_ and 4Gy in A549 cancer cells. **(C)** mRFP-GFP-LC3B are used in A549 cancer cells when treated with 1α,25(OH)_2_D_3_ and 4Gy. **(D)** Representative images of xenograft tumors sections stained for LC3. The data represents the Mean ± SD, *p < 0.05, ****p < 0.0001, n=3.

Figure S4 The enhancing radiosensitivity of 1α,25(OH)_2_D_3_ in cancer cells by promoting senescence. The data represents the Mean ± SD, **p < 0.01, n=2.

Figure S5 Effects of 1α,25(OH)_2_D_3_ on different cancer cells by CCK-8 assay, such as **(A)** Mouse primary ovarian cancer cells, **(B)** OVCAR8 cells, **(C)** A549 cells and **(D)** SKOV3 cells. The data represents the Mean ± SD, *p<0.05, **p < 0.01, ***p < 0.001, n=3.
