# Supplementary figures and images for: 1α,25(OH)2D3 Radiosensitizes Cancer Cells by Activating the NADPH/ROS Pathway
Source: Front Pharmacol. 2020 Aug 7;11:945. doi: 10.3389/fphar.2020.00945 (PMC7426479; doi:10.3389/fphar.2020.00945)

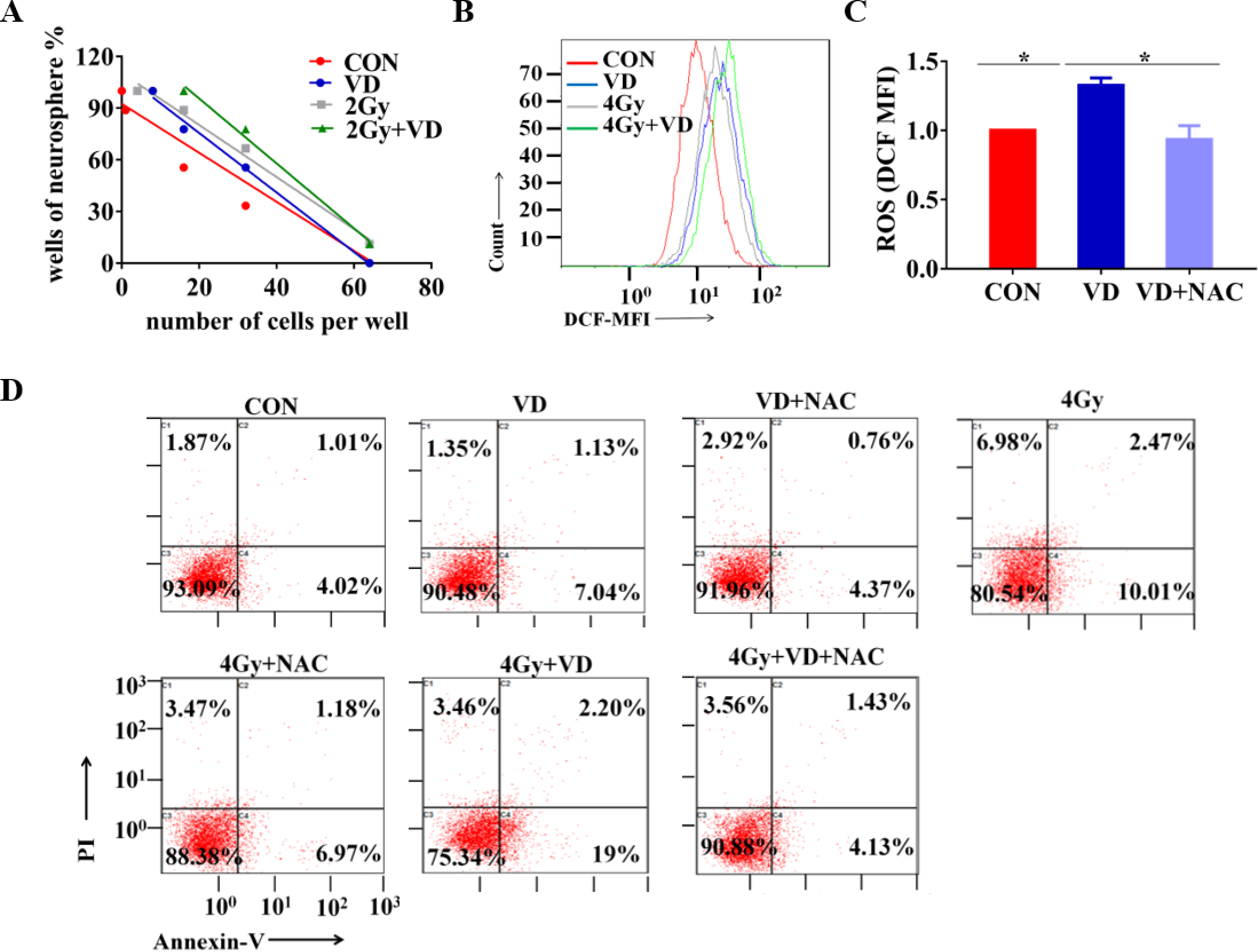

Supplement: Figure S1 — 1α,25(OH)2D3 enhanced the radiosensitivity of human ovarian cancer and lung cancer cells by ROS-induced apoptosis. (A) Results of limited dilution assay in SKOV3 cancer cells. (B) Effects of 1α,25(OH)2D3 and radiation on ROS of A549 cancer cells. (C) Effects of NAC to decrease the ROS generated by 1α,25(OH)2D3 in A549 cancer cells. (D) Combined effects of 1α,25(OH)2D3 and radiation on apoptosis of A549 cancer cells. Data represents the Mean ± SD, *p < 0.05, **p < 0.01, ***p < 0.001, n = 3. [file Image_1.tif]

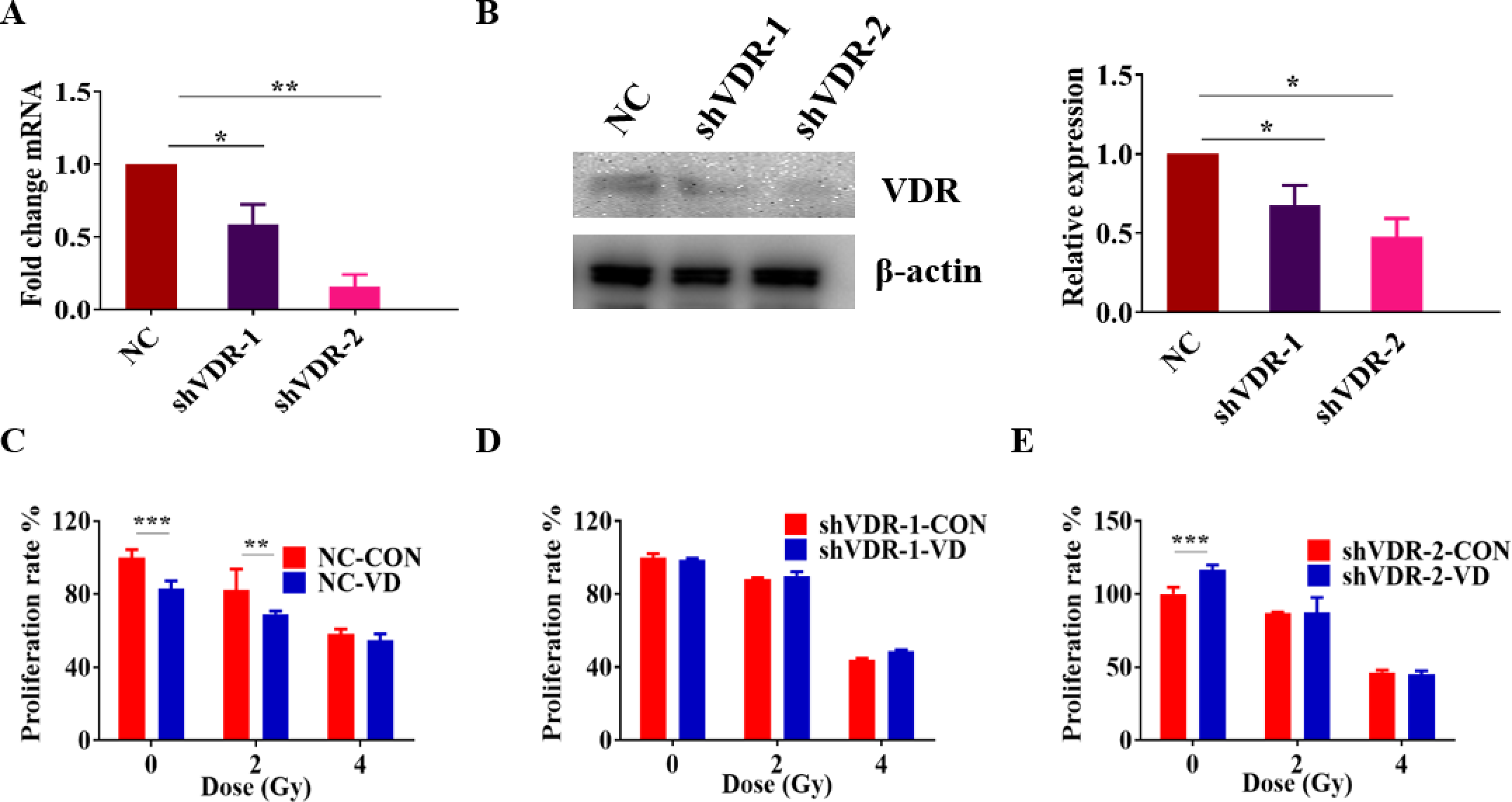

Supplement: Figure S2 — The enhancing radiosensitivity of 1α,25(OH)2D3 depends on VDR. (A, B) The mRNA and protein levels of VDR were examined when treated with shNC and shVDR in A549 cancer cells. (C-E) Colony formation assay was examined in A549 cancer cells when treated with shNC and shVDR. The data represents the Mean ± SD, * p < 0.05, ** p < 0.01, *** p < 0.001, n=3. [file Image_2.tif]

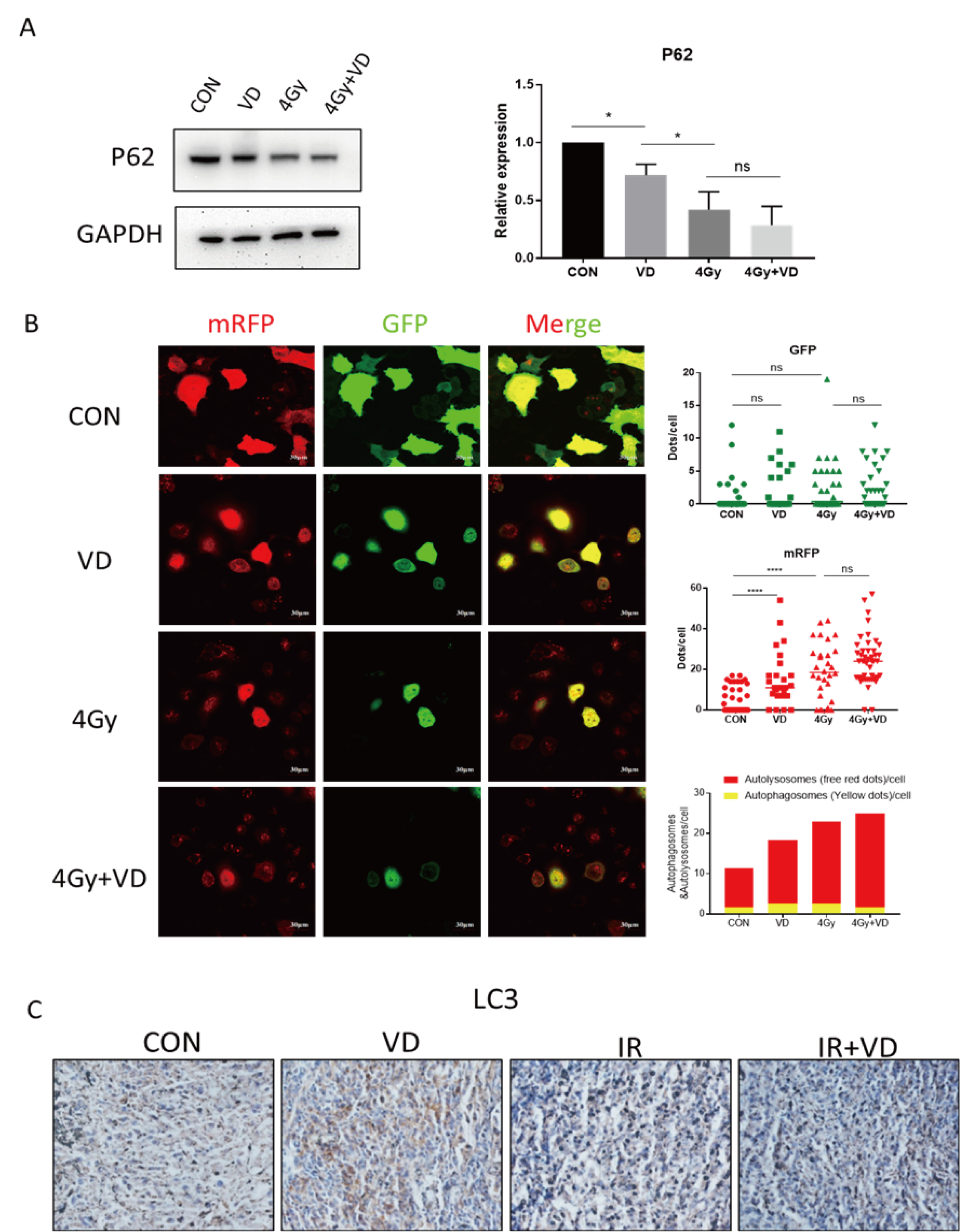

Supplement: Figure S3 — The enhancing radiosensitivity of 1α,25(OH)2D3 does not depend on autophagy. (A, B) The protein levels of P62 were examined when treated with 1α,25(OH)2D3 and 4Gy in A549 cancer cells. (C) mRFP-GFP-LC3B are used in A549 cancer cells when treated with 1α,25(OH)2D3 and 4Gy. (D) Representative images of xenograft tumors sections stained for LC3. The data represents the Mean ± SD, *p < 0.05, ****p < 0.0001, n=3. [file Image_3.tif]

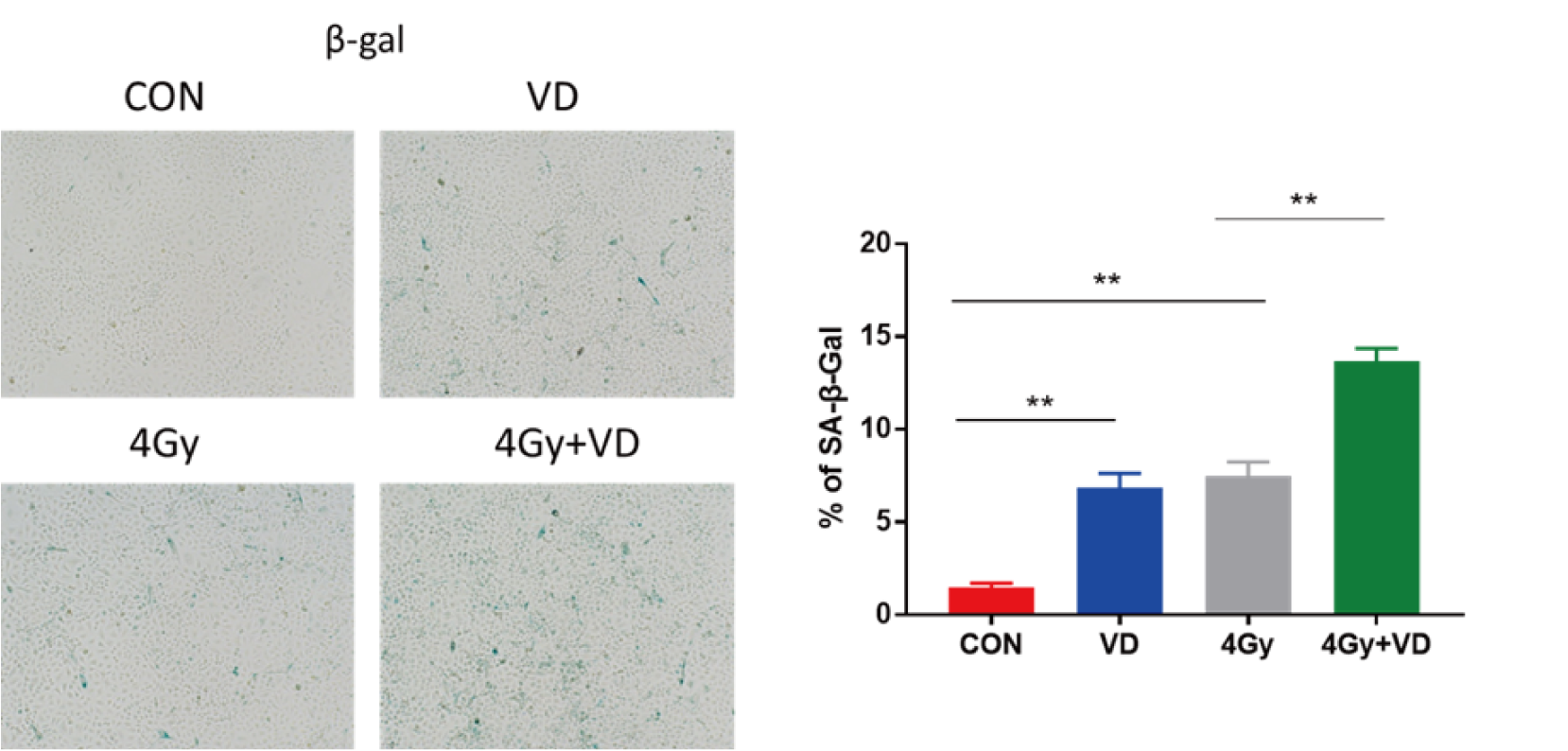

Supplement: Figure S4 — The enhancing radiosensitivity of 1α,25(OH)2D3 in cancer cells by promoting senescence. The data represents the Mean ± SD, **p < 0.01, n=2. [file Image_4.tif]

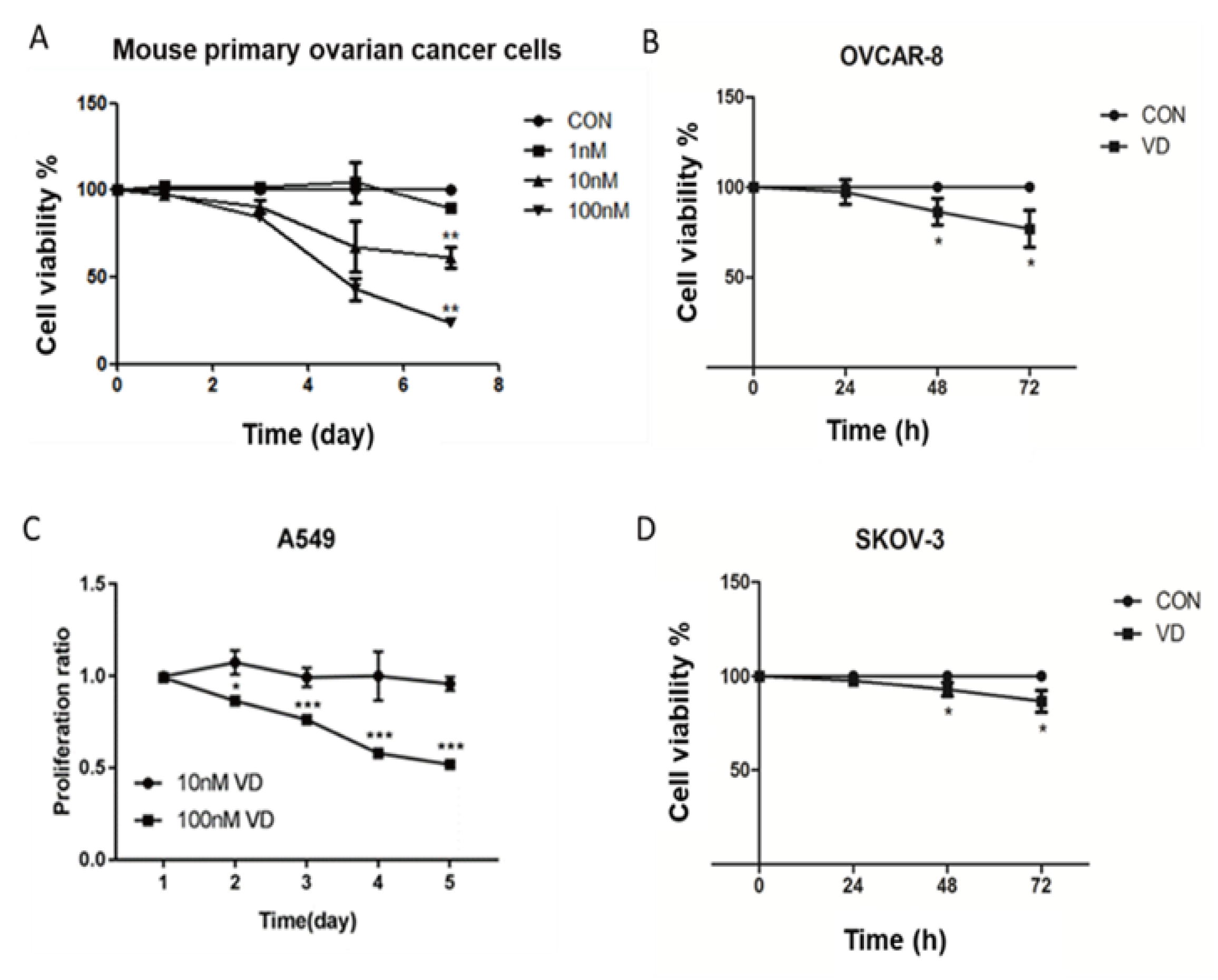

Supplement: Figure S5 — Effects of 1α,25(OH)2D3 on different cancer cells by CCK-8 assay, such as (A) Mouse primary ovarian cancer cells, (B) OVCAR8 cells, (C) A549 cells and (D) SKOV3 cells. The data represents the Mean ± SD, *p<0.05, **p < 0.01, ***p < 0.001, n=3. [file Image_5.tif]
